# Absence of food alternatives promotes risk-prone feeding of unpalatable substances in honey bees

**DOI:** 10.1038/srep31809

**Published:** 2016-08-18

**Authors:** Lucie Desmedt, Lucie Hotier, Martin Giurfa, Rodrigo Velarde, Maria Gabriela de Brito Sanchez

**Affiliations:** 1 Research Centre on Animal Cognition, Center for Integrative Biology, University of Toulouse; CNRS, UPS, 118 route de Narbonne, 31062 Toulouse cedex 09, France; 2 Departamento de Biodiversidad y Biología Experimental, Grupo de Estudio de Insectos Sociales, Facultad de Ciencias Exactas y Naturales, Universidad de Buenos Aires, Pabellón II, Ciudad Universitaria (C1428EHA), Buenos Aires, Argentina

## Abstract

The question of why animals sometimes ingest noxious substances is crucial to understand unknown determinants of feeding behaviour. Research on risk-prone feeding behaviour has largely focused on energy budgets as animals with low energy budgets tend to ingest more aversive substances. A less explored possibility is that risk-prone feeding arises from the absence of alternative feeding options, irrespectively of energy budgets. Here we contrasted these two hypotheses in late-fall and winter honey bees. We determined the toxicity of various feeding treatments and showed that when bees can choose between sucrose solution and a mixture of this sucrose solution and a noxious/unpalatable substance, they prefer the pure sucrose solution and reject the mixtures, irrespective of their energy budget. Yet, when bees were presented with a single feeding option and their escape possibilities were reduced, they consumed unexpectedly some of the previously rejected mixtures, independently of their energy budget. These findings are interpreted as a case of feeding helplessness, in which bees behave as if it were utterly helpless to avoid the potentially noxious food and consume it. They suggest that depriving bees of variable natural food sources may have the undesired consequence of increasing their acceptance of food that would be otherwise rejected.

Food is essential for survival but it can also be risky if it is associated with toxins that may endanger an animal’s life^1^. Yet, under certain circumstances, animals may accept noxious food, especially if their energy budgets are significantly low. Trade-offs between energetic needs and acceptance of noxious food have been shown both in vertebrates and in invertebrates^2^,^3^. For instance, *Drosophila melanogaster* larvae show more risk-prone feeding of noxious food (a liquid diet containing sugar and yeast paste, adulterated with 0.5% quinine) with longer deprivation periods whilst non-deprived larvae exhibit strong rejection of the same food^3^. Similarly, marine snails *Pleurobranchaea californica*, which normally reject the noxious aminoacid derivative taurine, exhibit feeding responses to taurine if in a hunger state^4^. Also, European starlings (*Sturnus vulgaris*) increase their attempts of eating chemically defended insect larvae when their body mass and fat stores are experimentally reduced^2^.

Honey bees are standard models for the study of olfactory^5^,^6^,^7^ and visual perception^8^,^9^,^10^,^11^,^12^. Yet, despite the economic relevance of their feeding behaviour in pollination and apicultural industries, important aspects of their feeding biology remain unknown^13^,^14^. For instance, the capacity of bees to recognize toxins is controversial^15^,^16^,^17^. In nature, nectars containing unpalatable substances such as amygdalin, caffeine and phenolics are, in fact, preferred by honey bees although if concentrations of such substances are too high, nectars are usually rejected^18^,^19^,^20^. In the laboratory, harnessed bees ingest without reluctance different kinds of noxious substances, including bitter ones, and even die as a consequence of the malaise induced by this ingestion^21^. On the contrary, free-flying bees trained to solve visual discriminations avoid bitter substances used as a penalty^22^,^23^,^24^. A possible explanation for this discrepancy may be that the avoidance of noxious foods depends on energy budgets. Indeed, laboratory experiments with harnessed bees usually subject these insects to starvation in order to increase appetitive responses^25^. Under these conditions, bees may be prone to accept noxious food. On the contrary, bees used in visual discrimination experiments fly freely between the hive and the experimental setup and are not subjected to starvation^26^.

An alternative explanation may be that these two experimental scenarios differ in the possibility left to the bees to sample safe vs. toxic food and to avoid, therefore, the latter. While free-flying bees can perform such a comparison by flying from a distracter punished with an aversive solution to a target rewarded with sucrose solution, harnessed bees fed uniquely with toxic food have neither escape nor an alternative for comparison; they may, therefore, behave as if it were utterly helpless to avoid the toxic food and consume it.

We asked whether noxious-food avoidance in honey bees depends on their energy budget or on the possibility of choosing between foods of different quality. We subjected late-fall/winter bees to two different feeding regimes in order to vary their energy budget. We then measured the mortality induced by different mixtures of sucrose solution and a noxious/unpalatable substance, in order to determine their toxicity. Finally, we compared mixture consumption of starved and fed bees when they had no alternative feeding option and when they could choose between a mixture of sucrose solution and a noxious substance and a pure sucrose solution. Our results reveal that the availability of food choices is more determinant of the bees’ feeding behaviour than their energy budget: in the absence of better, alternative food sources, fed and starved bees abandon their food preferences and consume non-preferred food, providing that it did not induce high mortality. These results raise important questions about agricultural practices and the food offer that such practices create for pollinators.

## Results

### Mortality after ingestion of mixtures of sucrose solution and unpalatable solutions

In a first experiment, we determined the mortality induced by the solutions used in our treatments using a Kaplan-Meier survival analysis. In doing this experiment, our concern was not the quantification of the sublethal effects induced by these substances, but establishing whether they were or not toxic, or just unappetizing but not necessarily toxic.

Bees were caught in the morning at the entrance of a hive located at 50 m from the laboratory and harnessed in individual tubes, fed with 0.6 M sucrose solution and kept in the dark and in high humidity for approximately three hours, a period that is sufficient to induce starvation and higher sucrose responsiveness^27^. Harnessing was necessary as under these experimental conditions, bees are prone to ingest pure noxious substances delivered by means of a graded micropipette^21^. Different groups of bees were fed 20 μl of either 0.6 M sucrose (n = 19), 0.6 M sucrose + 100 mM salicin (n = 19), 0.6 M sucrose + 10 mM quinine (n = 20) or 0.6 M sucrose + 3 M NaCl (n = 16). Salicin, quinine and NaCl were chosen as they were shown to induce significant mortality when fed alone at these concentrations^21^.

**Figure 1:**
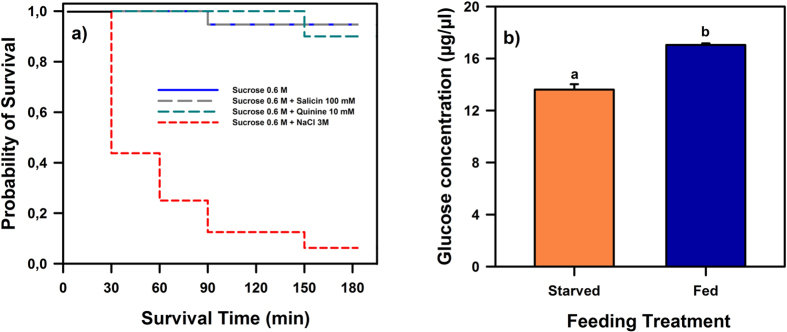
(**a**) Kaplan–Meier curves of survival for harnessed honeybees following ingestion of four feeding treatments. The probability of survival differed significantly between groups. Bees that ingested a mixture of 0.6 M sucrose solution and 3 M NaCl (n = 16) exhibited highest mortality so that their probability of survival decreased following ingestion; on the contrary, ingestion of mixtures of 0.6 M sucrose and 10 mM quinine (n = 20) or 100 mM salicin (n = 19) had no significant impact on mortality as survival did not differ from that observed after ingestion of sucrose 0.6 M alone (n = 19). **(b)** Starved and fed bees differed in their energy budget. Glucose concentration in the haemolymph of bees that received different feeding treatments: the group ‘Fed’ (n = 80) had access to sucrose solution 0.6 M while the group ‘Starved’ (n = 80) had only access to water *ad libitum.* Bees remained in their respective cage for 21 h, thus totalling 24 h since their capture. The figure shows the glucose in haemolymph (mean ± S.E.; μg/μl) measured at the end of the 24-h period. The concentration of glucose of the group ‘Fed’ was significantly higher than that of the group ‘Starved’, thus revealing that the two groups had significantly different energy budgets. Different lower-case letters above bars indicate significant differences (p < 0.0005).

We quantified the number of dead bees at 30, 60, 90, 120, 150 and 180 min following feeding of the last bee in a group. For each treatment (i.e. solution fed), we computed the cumulative proportion of surviving bees and established Kaplan-Meier’s survival functions defined as the probability of surviving at least to 3 h^21^. Figure 1a shows that despite the fact that bees ingested all four solutions, the probability of survival differed significantly between groups (log-rank test: χ^2^ = 54.33, df:3, p < 0.0001). Bees that ingested a mixture of 0.6 M sucrose solution and 3 M NaCl (n = 16) exhibited highest mortality so that their probability of survival decreased dramatically following ingestion; on the contrary, ingestion of mixtures of 0.6 M sucrose and 10 mM quinine (n = 20) or 100 mM salicin (n = 19) had no significant impact on mortality as survival did not differ from that observed after ingestion of 0.6 M sucrose alone (χ^2^ = 0.36, df:2, p = 0.84). These results thus show that from the substances assayed, only the mixture of 0.6 M sucrose and 3 M NaCl had a noxious effect. Although quinine and salicin solutions induce significant mortality when ingested alone^21^, their mixing with sucrose solution suppressed their noxious effect and supported survival during the 3 h following ingestion.

### Consumption of non-preferred food by starved and fed bees: energy budgets

To determine whether avoidance of non-preferred food in honey bees depends on their energy budget or on the possibility of choosing between foods of different quality, we subjected bees to two different feeding regimes in order to vary their energy budget. Bees were caught in the morning at the entrance of a hive located at 50 m from the laboratory and placed in small cages (8,5 cm × 5 cm × 4 cm) where they had access to a mixture of honey, pollen, sucrose and water during 3 h in order to homogenize their nutritional state. Afterward, bees were randomly assigned to two groups, which received different feeding treatments during 21 h: the 1^st^ group had access to 0.6 M sucrose solution (henceforth ‘fed’; n = 600) prepared with distilled water, while the 2^nd^ group had only access to distilled water *ad libitum* (henceforth ‘starved’; n = 524).

To verify that the two feeding treatments resulted in different energy budgets, we measured for 20 bees of each group the glucose concentration in the haemolymph at the end of the 21-h period. Four replicates were performed for this measurement (i.e. n = 160, 80 for each group). Figure 1b shows that the concentration of glucose (μg/μl) of the group fed with 0.6 M sucrose solution was significantly higher than that of the starved (water-fed) group (t-test for independent samples: t_6_ = 8.02, p < 0.0005), thus confirming that the two groups had significantly different energy budgets at the end of their respective treatments. In the next two experiments, the same procedure was therefore used to establish groups of caged bees, which had different energy budgets at the end of the 21-h and which were afterwards offered different toxic and/or unpalatable solutions.

### Consumption of non-preferred food by starved and fed bees: grouped bees in a dual-choice situation

As with the previous experiment, honey bees were caught in the morning at the entrance of a hive located at 50 m from the laboratory. They were then placed in small cages (8,5 cm × 5 cm × 4 cm) where they had access to a mixture of honey, pollen, sucrose and water during 3 h in order to homogenize their nutritional state (Supplementary Fig. 1a). Bees were afterwards randomly assigned to two groups, which received different feeding treatments during 21 h: the 1^st^ group had access to 0.6 M sucrose solution (henceforth ‘fed’) prepared with distilled water, while the 2^nd^ group had only access to distilled water *ad libitum* (henceforth ‘starved’).

On the second day, groups of caged bees, fed and starved, were presented during 3 h with a dual-choice situation via two Eppendorf tips containing different foods (1 ml of 0.6 M sucrose solution vs. 1 ml of the same solution mixed with a potentially noxious substance, 10 mM quinine, 100 mM salicin or 3 M NaCl; Supplementary Fig. 1a). An independent group of bees was used for each dual-choice situation (i.e. 6 groups in total; 3 starved, four to five replicates of each (n = 255) and 3 fed, four to five replicates of each (n = 259 bees). Bees could thus choose between two alternative feeding options, one appetitive and the other non-appetitive, in an assay that was reminiscent of the Capillary Feeder (CAFE) assay, a method allowing precise measurement of ingestion by grouped insects^28^. We recorded the average consumption of the substances offered, at the end of the 3-h test. Consumption values were corrected for evaporation, which was measured in control cages^29^ without bees, and expressed in terms of μl consumed per min per bee to facilitate comparisons between experiments.

**Figure 2:**
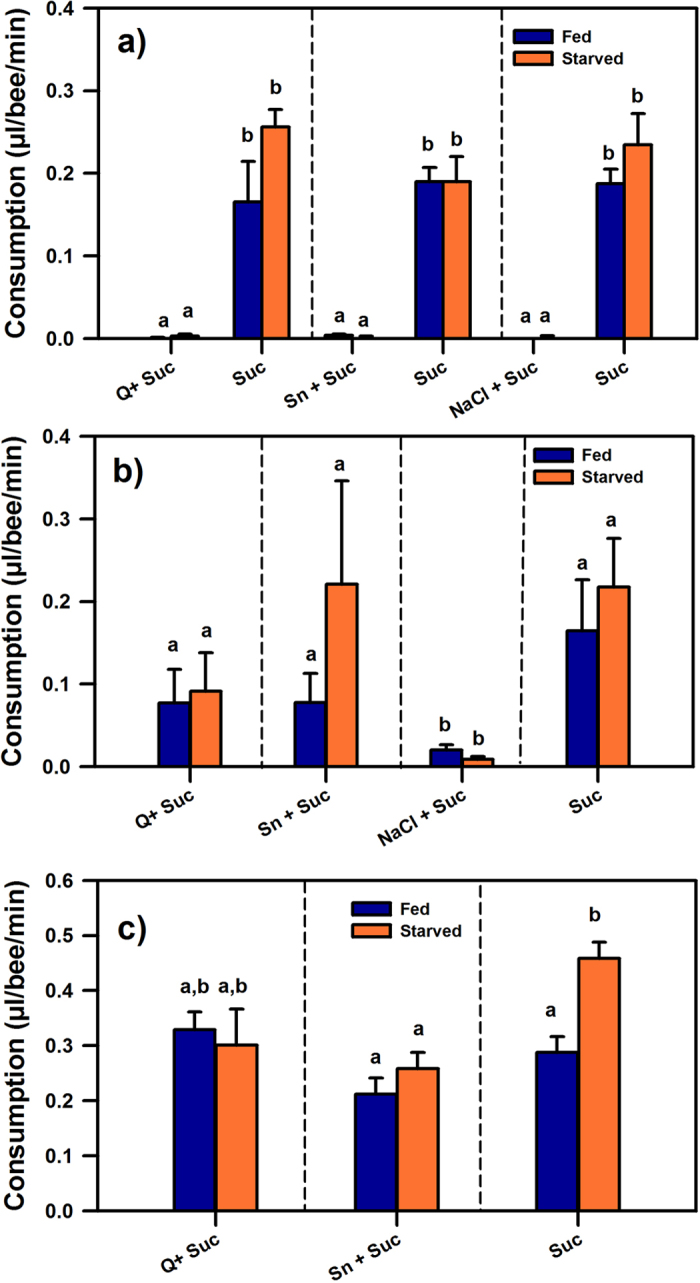
(**a**)Food consumption by groups of bees in a dual-choice situation. Consumption (μl/min/bee; mean ± S.E.) was measured 3 h after offering one of the three dual-choice situations (Suc vs. Q + Suc: 0.6 M sucrose solution vs. a mixture of 0.6 M sucrose solution and 10 mM quinine; Suc vs. Sn + Suc: 0.6 M sucrose solution vs. a mixture of 0.6 M sucrose solution and 100 mM salicin; Suc vs. Nacl + Suc: 0.6 M sucrose solution vs. a mixture of 0.6 M sucrose solution and 3 M NaCl). Three groups of starved bees (four to five replicates of each; n = 255) and 3 of fed bees (four to five replicates of each; n = 259) were used. Both starved (orange bars) and fed bees (blue bars) preferred the pure sucrose solution to any mixture. There were no significant differences between starved and fed bees for all three dual choices. **(b)** Food consumption by groups of bees in a single-choice situation. Consumption was measured 3 h after offering one of four food options. Four groups of starved bees (four replicates of each; n = 108) and 4 of fed bees (four replicates of each; n = 167 bees) were used. Both fed (blue bars) and starved bees (orange bars) consumed significantly less of the toxic mixture (Nacl + Suc) and accepted solutions that they rejected when choice was available (Q + Suc and Sn + Suc). There were no significant differences between fed and starved bees. **(c)** Consumption of single food options by isolated bees. Consumption was measured 2 h after offering one of three food options. Three groups of fed bees (n = 98) and three groups of starved bees (n = 76) were used. The consumption of the mixtures Q + Suc and Sn + Suc did not vary between fed (blue bars) and starved bees (orange bars) and was higher than that of caged bee groups (compare with Fig. 2b). In all three panels, different lower-case letters above bars indicate significant differences (Tukey tests, p < 0.05).

Both starved and satiated bees preferred the pure sucrose solution to a mixture of sucrose solution and quinine, salicin or NaCl (Fig. 2a). Fed bees (Fig. 2a, blue bars) preferred the pure 0.6 M sucrose solution to any of the three mixtures (two-way repeated measurement ANOVA; factor choice: F_1,10_ = 108.49, p < 0.00001) and this preference was the same irrespectively of the alternative mixture offered (factor solution: F_2,10_ = 0.22, p = 0.81). The interaction between choice and solution was not significant, showing that the pattern of responses was the same for all three dual-choices (F_2,10_ = 0.18, p = 0.84). Starved bees showed the same pattern of responses (Fig. 2a, orange bars); they preferred the pure 0.6 M sucrose solution to any of the three mixtures (factor choice: F_1,10_ = 138.91, p < 0.00001) and this preference was the same independently of the alternative mixture offered (factor solution: F_2,10_ = 1.09, p = 0.37). The interaction between choice and solution was also not significant (F_2,10_ = 0.94, p = 0.42). Importantly, there were no significant differences between starved and fed bees for all three dual choices (F_1,20_ = 3.34, p = 0.08), thus showing that the bees’ energy budget did not affect their feeding responses: they always preferred the pure sucrose solution and avoided the mixtures of sucrose and quinine, salicin or NaCl. No differences in avoidance were found between these three mixtures (post hoc Tukey tests: NS for all three comparisons), even if only one of them (sucrose + NaCl) was truly toxic (see above). At the end of the 3 h-measurement period, bees had ingested an average amount of 36.70 μL (±2.51; S.E.) of pure sucrose solution, which represents 61% of the full crop capacity (60 μl in average) of an European bee^30^, and only 0.32 μL (±0.10) of the mixtures.

### Consumption of non-preferred food by starved and fed bees: grouped bees in a single-choice situation

In parallel to the previous experiment, we measured food consumption in another set of caged fed and starved bees when both tips contained the same solution (either 1 ml of 0.6 M sucrose solution or 1 ml of the same solution mixed with 10 mM quinine, 100 mM salicin or 3 M NaCl). Bees were handled as before, but in this case, and contrary to the prior dual-choice situation, they had no alternative choice except to consume or not the only food available (Supplementary Fig. 1b). An independent group of bees was used for each situation (i.e. 8 groups in total; 4 starved, four replicates of each, n = 108; 4 fed, four replicates of each, n = 167 bees). As with the previous experiment, we recorded the average consumption of the substances offered, at the end of the 3-h test and consumption values were corrected for evaporation^29^, and expressed in terms of μl consumed per min per bee to facilitate comparisons between experiments.

Figure 2b shows that satiated bees consumed all four feeding options at a similar level despite a tendency to consume less the toxic mixture of 0.6 M sucrose and 3 M NaCl (one-way ANOVA for independent groups; F_3,12_ = 2.09, p = 0.16). Starved bees showed a similar pattern of responses as their consumption did not differ between feeding options (F_3,12_ = 1.96, p = 0.17) even if the consumption of the mixture of sucrose 0.6 M and NaCl 3 M was again lower. A comparison between fed and starved bees showed that there were not significant differences in consumption according to the satiety state (two-way ANOVA for independent groups; factor satiety: F_1,24_ = 1.45, p = 0.24), but differences between solutions appeared now in this global analysis (factor solution: F_3,24_ = 3.34, p < 0.05) showing that both starved and fed bees consumed significantly less of the toxic mixture of 3 M Nacl and 0.6 M sucrose (Tukey tests: p < 0.05). All in all, although starved and fed bees showed a similar feeding behaviour when facing a unique feeding option, they clearly accepted solutions that they rejected when they had the opportunity to choose, in particular the mixtures of sucrose and quinine or salicin. Indeed, mixture consumption varied significantly between the dual-choice and the single-choice experiments (F_1,38_ = 12.51, p < 0.001) because the consumption of the mixtures of sucrose and quinine and sucrose and salicin increased in two orders of magnitude in the single-choice experiment (p < 0.05 for both mixtures). On the contrary, the consumption of pure sucrose solution remained stable between both experiments as at the end of the 3 h-measurement period, bees had ingested an average amount of 34.39 μL (±4.78) of pure sucrose, which correspond to 57.32% of the full crop capacity^30^ and coincides with the 61% level found in the previous experiment.

### Consumption of non-preferred food by starved and fed bees: individually-isolated bees in a single-choice situation

In a further experiment, we aimed at measuring food consumption in a single-choice situation with increased stressful conditions. To this end, we confined bees individually in the reduced space of a 5 mL syringe where their movements were restricted and where contrary to the previous experiments, no social interactions existed. This experiment was important as an increased consumption of non-preferred food by groups of caged bees (Fig. 2b) could have been due to social facilitation rather than to an individual decision of consuming more of a non-preferred food, when no other choice is available. Furthermore, the experimental conditions were more stressful as bees were deprived of social contacts. Owing to these restrictive conditions, the timing of the experiment phases could not be the same as in the two previous experiments with caged bees (Supplementary Fig. 1c). Confinement within the syringe had thus to be limited to reduce mortality, which otherwise would be too high.

At the beginning of the experiment, 1 mL Eppendorf tip containing honey was inserted in the syringe tip so that the bee could access the food, which was generally consumed after 5 min. Bees were then divided according to the two previous feeding treatments (Supplementary Fig. 1c): fed bees (n = 98) received sucrose solution 0.6 M prepared in distilled water, delivered by a 1 mL Eppendorf tip inserted in the syringe during a 4-h period. Starved bees (n = 76) received distilled water during the same period using the same method. The 4-h duration of this phase was chosen after confirming that longer periods of isolation induced excessive mortality in restrained bees^31^,^32^,^33^.

After the end of the 4-h period, fed and starved bees were divided in three subgroups (six in total) and presented during 2 h with a unique feeding option within their syringe via a new 1 mL Eppendorf tip containing either a pure 0.6 M sucrose solution, a mixture of 0.6 M sucrose solution and 10 mM quinine, or a mixture of 0.6 M sucrose solution and 100 mM salicin. The mixture of sucrose solution and NaCl was not assayed as no variations in its consumption were found between the dual-choice and the single choice experiments with caged bees (see above). The food-exposure period was also reduced compared to previous experiments (from 3 to 2 h) for the reasons discussed above. Consumption values at the end of the 2-h period were corrected for evaporation^29^, and expressed in terms of μl consumed per min per bee.

Figure 2c shows the results of individual food consumption of fed and starved bees for the three feeding treatments. A global comparison between fed and starved bees showed that there were again no significant differences in consumption according to the satiety state of the bees (two-way ANOVA for independent groups; factor satiety: F_1,169_ = 3.59, p = 0.06) but a clear difference between solutions could be observed (factor solution: F_3,163_ = 11.17, p < 0.0001). Post-hoc Tukey tests revealed that this difference was introduced by the starved bees, which were offered the pure sucrose solution. These bees consumed significantly more sucrose than the fed bees presented with sucrose and salicin and the starved bees presented with salicin (p < 0.01 for all 3 comparisons). Importantly, the rate of consumption of the mixtures of sucrose and quinine and sucrose and salicin did not vary between fed and starved bees and tended to be twice as high as that recorded in the single-choice experiment with the caged groups of bees (compare with Fig. 2b: F_1,105_ = 3.73, p = 0.06; note the different scales of the ordinate axes). Furthermore, at the end of the 2 h-food exposure period, isolated bees ingested 28.22 (±2.78) and 37.80 μL (±1.70) of the mixtures of sucrose and salicin and sucrose and quinine, respectively, which correspond to 47.03% and 63% of their full crop capacity^30^, and thus coincide with the amounts of pure sucrose solution typically ingested in the caged experiments (see Fig. 2a,b). These results clearly show that social facilitation does not account for the increase in the consumption of these non-appetitive substances. The more restrictive enclosing conditions could have enhanced the bees’ proneness to ingest the less attractive food, when no other choice was available. All in all, this result confirms that the feeding behaviour of bees was mainly determined by choice availability and isolation stress rather than by energy budget.

## Discussion

### Energy budget and availability of alternative food sources

Our study contrasted two different hypotheses to explain noxious food acceptance in adult honey bees: the individual’s energy budget and the availability of an alternative feeding choice. While the former posits that energy-depleted individuals are more risk-prone in food acquisition than those on a positive energy budget^3^,^4^,^34^, the latter maintains that when individuals have neither escape nor an alternative feeding choice, they may consume noxious food. From these two hypotheses, the latter provided a full account of the feeding behaviour of late-fall and winter bees and was, therefore, the main driving factor of the bees’ choices in our experiments. However, the levels of significance found when comparing fed and starved bees in the experiments with groups of bees in a dual-choice situation (Fig. 2a) and with individually-isolated bees in a single-choice situation (Fig. 2c) were both marginally non-significant (p = 0.08 and p = 0.06, respectively). Thus, even if choice availability was the main factor in our experiments, a minor role of the bees’ energy budget cannot be totally excluded. This conclusion is confirmed by the fact that when bees were set in isolation and presented with pure sucrose solution, a difference was observed as starved bees consumed more sucrose (Fig. 2c). As this effect was not observed in the case of the other solutions tested in the same experimental conditions, the energy budget did not provide an integrative account of our results. On the contrary, choice availability changed significantly the feeding behaviour of the bees: whenever they had the opportunity to choose and sample alternative feeding options, bees avoided non-appetitive substances, irrespectively of their energy budget (Fig. 2a). In contrast, when no alternative choice was offered, the bees consumed the non-appetitive substances and abandoned their original feeding preferences (Fig. 2b). We do not exclude that with longer starvation periods (>21 h), the energy budget becomes more determinant for hungry honey bees; yet, starvation periods of 21 and 24 h have proved to be sufficient to generate differences in other contexts both at the behavioural and neurotransmitter levels^15^,^35^,^36^,^37^. Moreover, longer starvation periods result in high mortality, thus rendering the question difficult to address experimentally.

Another type of explanation for the lack of a full effect of starvation on the consumption of non-preferred food may revolve around the particular bees used in these experiments. Late-fall and winter bees captured at the hive entrance were used, which differ physiologically from springtime and summer bees. Winter bees exhibit lower levels of juvenile hormone, high vitellogenin titers, active fat body and high nutritional stores in general^38^,^39^,^40^,^41^. It may be, therefore, that in winter bees hunger is not as determinant of noxious food consumption as in summer bees in which starvation could promote noxious food consumption *per se*. For the autumn and winter bees used in our experiments, feeding was mostly determined by choice availability despite the fact that significant differences in energy budget were detectable via measurements of glucose concentration in the haemolymph. More studies are necessary to determine if winter/autumn and summer/springtime bees differ in their responses to unpalatable sugar sources.

### Absence of food consumption does not always reflect food toxicity

Our results underline the necessity of careful analyses before labelling a substance as toxic. It is usually assumed that substances that taste bitter to humans are also bitter to other animals, including insects, without considering that animals may differ from humans in their gustatory-receptor repertoire and in their capacity to sense bitter taste and toxins. Our results show that there is a clear difference between mixtures of sucrose solution and substances that taste bitter to humans such as quinine and salicin on one hand, and a mixture of sucrose solution and NaCl on the other hand. While the latter was definitely toxic as it induced significant mortality after being ingested, the former did not induce mortality (Fig. 1b) and were therefore not noxious despite of previous reports mentioning a supposed toxicity of mixtures of sucrose and bitter substances e.g. ref. 42. In both cases, rejection was observed when bees had the opportunity to choose between a mixture and a pure sucrose solution; yet, the reasons for such rejection may be different. While a mixture of sucrose and NaCl may be indeed distasteful owing to its toxicity and physiological effects, a mixture of sucrose and quinine or salicin may be rejected due to a reduction or lack of sweet taste (i.e. due to its unappetizingness), rather than to a repulsive nature. Electrophysiological studies have shown that chaetic sensilla on the antennae and the tarsi that respond to sucrose are inhibited by stimulation with a mixture of sucrose and quinine^16^,^17^,^43^,^44^,^45^, and the molecular mechanism accounting for this inhibition has been recently described in the fruit fly^46^. It is thus possible that such a mixture could be perceived, not as a distasteful substance, but rather as a non-sweet aqueous substance. This possibility would explain why bees accept to feed on mixtures of sucrose and bitter substances and less on a mixture of NaCl and sucrose solution when no choice is available (Fig. 2b): only the latter would be truly distasteful and aversive while the former could still be accepted, in particular if sucrose masks in part the bitter taste^45^. A similar interpretation may apply to reports mentioning that some bees do not drink sucrose solution with a quinine concentration of 10 mM or higher after having been stimulated with pure concentrated sucrose solution on the antennae to extend the proboscis^15^: in this case, rejection may be due, not to a distasteful nature of the mixture, but to its failure to fulfil the expectation of a true sweet reinforcement induced by sucrose solution on the antennae. Survival analyses (Fig. 1a) support this hypothesis as the presence of sucrose compensates the potential toxic effect that substances such as quinine or salicin may have on their own, when fed in a pure state^21^. In the case of NaCl, on the contrary, such compensation does not occur.

### Feeding helplessness as a determinant of food consumption

The feeding behaviour of bees confronted with a unique feeding choice resembles a case of ‘helplessness’^47^ as bees fed uniquely with pure noxious substances^21^ or non-appetitive mixtures of sucrose and noxious substances (this work) have neither escape nor an alternative for comparison; they may, therefore, behave as if it were helpless to avoid the noxious or less attractive food and consume it. This interpretation is supported by the tendency to increase the consumption of non-appetitive solutions when restraining conditions were accentuated by isolation in an enclosing syringe (Fig. 2c). In this case, bees ingested volumes of sucrose and quinine and sucrose and salicin that were higher than when they were caged as a group and confronted with the same food, even if in the isolation experiment consumption was measured only after a 2-h period while in the group experiment after a 3-h period. Note that although the timing of the two experiments was different, the fact that consumption values were normalized by unit time supports the interpretation that the stressful conditions imposed by individual confinement enhanced consumption of the non-preferred mixtures.

Learned helplessness was first attributed to dogs that showed a failure to avoid an electric shock when given the opportunity, after previous exposure to inescapable shocks^48^,^49^. In fruit flies, learned helplessness has been shown using a heat punishment^50^. In this case, a fly heated as soon as it stops walking resumes walking to escape the heat; if the fly is not in control of the heat, it starts walking slowly and taking longer and more frequent rests. In bees, experiments on learned helplessness using electric shocks are difficult as these insects do not exhibit freezing to an inescapable electric shock^51^.

We suggest that the feeding behaviour of bees constrained to feed on a unique aversive food constitutes an example of this learned helplessness. In our experiments, the acceptance of non-appetitive, yet non-toxic substances (mixtures of sucrose solution and quinine or salicin) when there was no feeding alternative, constitutes an example of how feeding behaviour can be adjusted to the situation experienced as the same substances are rejected when better choices are available. This strategy did not extend, however, to the mixture of sucrose and NaCl, which was truly toxic. Thus, although feeding behaviour is adaptable, the necessity to survive sets the limits for this adaptability.

Our findings account for differences observed in the feeding behaviour of honey bees in studies varying in the possibility left to these insects to sample alternative food sources. In the laboratory, harnessed bees have been shown to ingest without reluctance different kinds of noxious substances, including bitter ones, and even die as a consequence of the malaise induced by this ingestion^21^. On the contrary, free-flying bees trained to solve visual discriminations avoid bitter substances used as a penalty^22^,^23^,^24^. This discrepancy remained unsolved until now and raised questions about the bees’ capacity to detect or not bitter substances. Our results indicate that risk-prone feeding in harnessed bees may be due to a large extent to the restraining conditions to which these bees are subjected, which would enhance their feeding helplessness and reduce, therefore, their acceptance thresholds for unpalatable and noxious food. On the contrary, bees used in visual discrimination experiments fly freely between the hive and the experimental setup and maintain their possibility of avoiding undesirable food^26^, thereby maintaining high and selective acceptance thresholds.

### Mechanisms of food-ingestion regulation via feeding helplessness

The energy budget hypothesis, which was not decisive for our results, accounts for the feeding behaviour of *Drosophila* larvae, which are more prone to feed on foods adulterated with 0.5% quinine with longer deprivation periods^3^. This risk-prone feeding relates to a neuropeptide signalling pathway involving one NPY-like neuropeptide (neuropeptide F, NPF) whose action is mediated by a protein-coupled receptor NPFR1. In the larvae, the propensity to feed on potentially toxic food correlates with higher levels of expression of NPFR1. Up-regulation of this receptor is sufficient to trigger intake of noxious food in non-deprived larvae. Conversely, disruption of neural NPFR1 signalling in deprived larvae leads to a decrease in noxious-food feeding^3^. In the honey bee, two NPY-related genes were identified, *npf* and *snpf*^52^, but only a receptor for the short (s) peptide (*snpf*R) was found^53^. One of us (R. Velarde) showed that *snpf*R is up-regulated in the brain of older foragers and these changes were attributed to nutrition as *snpf*R is up-regulated by food deprivation^54^. Besides the feeding-state dependency of *snpf*R expression, we suggest that feeding helplessness may also induce significant variations of *snpf*R expression in honey bees. We predict that bees subjected to a unique choice will exhibit higher levels of *snpf*R in their brains while bees having the possibility of overtly avoiding non-appetitive substances and choosing appetitive ones will exhibit lower levels of this receptor gene. This hypothesis posits, therefore, that the potential stress associated to the impossibility of avoiding noxious food will up-regulate *snpf*R expression, thus promoting the intake of less appetitive food.

### Honeybee feeding helplessness in colony and ecological context

Our results might have a broad ecological and conservational implication in the perspective of the massive loss of honey bee colonies reported in the last years^55^,^56^. It has been suggested that the decline of honeybees seen in many countries may be caused by reduced plant diversity resulting from intensive monoculture practice^57^,^58^. As a consequence, bees would be increasingly exposed to feeding situations in which the possibility of choosing alternative food sources is dramatically reduced. This may increase their acceptance of food that would be otherwise rejected, thereby affecting dramatically their survival.

Another possible scenario for our findings takes into account that the bees used in our experiments were essentially winter bees, which rarely leave the hive. These bees rely on colony honey stores for hive thermoregulation as floral nectar is scarce outside the hive. Such stores may include honey with unpalatable and even toxic substances. Our caged-bee experiments could mimic interactions within the colony where the bulk of sugar consumption occurs during prolonged dearth periods such as winter. The preference of caged bees for palatable sugar sources over unpalatable alternatives implies that palatable sugar stores would be consumed first in the hive. In the absence of incoming nectar, this situation would lead to a faster depletion of palatable relative to unpalatable/noxious food stores over time. Thus, unpalatable food stores would be primarily consumed towards the end of dearth or winter periods, when the colony is possibly attempting to increase brood production. This scenario could dramatically impact the development and survival of colonies that have gathered chemically toxic nectars in agricultural landscapes.

Although both scenarios are plausible, they need to be considered with caution as the experimental situations of our study are very different from natural ones. Further experiments at the colony and field scales are necessary to test if, as suggested, agricultural practices result in feeding helplessness and noxious food consumption in honeybees, either in a foraging or in a colony context.

## Methods

Honeybees, *Apis mellifera*, were caught in the morning, at the entrance of a hive located at 50 m from the laboratory. Experiments were performed during autumn and winter days in which bees were seen at the hive entrance due to the mild temperatures existing in the region of Toulouse. Captured bees were handled differently depending on the experiment.

### Survival measurements

To determine whether the substances to be fed in our experiments were toxic, or unpalatable but not necessarily toxic, we measured the probability of survival following substance ingestion by harnessed bees. Harnessed bees were used in this experiment as it has been shown that in these contention conditions bees ingest all kinds of substances, including those that they would eventually reject in normal conditions^21^. Our goal was thus to determine the acute effects of our feeding treatments and not their sub-lethal effects.

Captured bees were placed in glass vials and cooled down on ice until they stopped moving. They were then harnessed in individual small tubes so that they could only move their antennae and mouthparts, including the proboscis. Bees were then fed with 0.6 M sucrose solution and kept in the dark and in high humidity for approximately three hours, a period that is sufficient to induce starvation and high appetitive responsiveness^27^.

Different groups of bees (between 16 and 20 bees per group) were fed with different substances: 0.6 M sucrose, 0.6 M sucrose + 100 mM salicin, 0.6 M sucrose + 10 mM quinine and 0.6 M sucrose + 3 M NaCl. All chemicals were from Sigma – Aldrich (France). Within each group, each bee was fed 20 μl (4 times 5 μl; i.e. one third of their full crop load)^30^ of the substance assigned to its group. A graded micropipette was used to feed the bees so that we could verify the amount of solution ingested. Typically, the time needed to feed 5 μl of a substance to a bee was not longer than 20 s. Bees were fed one after the other until completing feeding of the group. Thus, the first round of feeding took approximately 400 s (i.e. 6 min) for 20 bees. As four rounds were necessary to complete feeding of the 20 μl, the time elapsed since the first and the last feeding of a bee was approximately 25 min; the delay between the 1^st^ and the last bee was – as explained – 6 min approximately (for a group with n = 20).

For each bee, survival was measured at 30, 60, 90, 120, 150 and 180 min post-feeding, taking as reference (t_0_) the time at which its last feeding took place. Thus, despite the short delay existing between the first and the last bee of a group, intervals for survival measurement were constant between bees. Survival analysis was performed using as censored observations the individuals that survived at the end of the measuring period^59^. For each treatment (i.e. solution fed), we computed the cumulative proportion of surviving and established Kaplan-Meier’s survival functions defined as the probability of surviving at least to time t. We used a log rank test to compare multiple samples, which is a standard procedure in survival analyses^59^. Such a log rank test follows a χ^2^ distribution in the case of multiple-sample comparison; in the case of two-sample comparisons, it computes a Z score referred to a normal distribution.

### Measurement of glucose levels in haemolymph

To verify that the experimental procedures aimed at creating fed and starved bees (see Supplementary Fig. 1a,b) resulted indeed in bees with different energy budgets, we measured the glucose concentration in the haemolymph of groups of 20 bees belonging to each group, at the end of their respective treatment.

Bees captured at the hive entrance were placed in small cages (8.5 cm × 5 cm × 4 cm) where they had access to a mixture of honey, pollen, sucrose and water during 3 h in order to homogenize their nutritional state. Cages were kept in cardboard box in the dark and at room temperature of 23 °C. Afterwards, they were divided in two groups subjected to different feeding treatments: a 1^st^ group had access to 0.6 M sucrose solution (henceforth ‘fed’) while a 2^nd^ group had only access to distilled water *ad libitum* (henceforth ‘starved’). Bees remained in their respective cage for 21 h, thus totalling 24 h since their capture.

At the end of this period, a total of 20 μl of haemolymph was obtained from 20 bees of each group (1 μl per bee). The haemolymph pool was then vortexed to mix thoroughly, and the glucose quantification was carried out. A glucose assay kit (Sigma Aldrich) was used to measure the glucose concentration in haemolymph following the manufacturer’s instructions. Four replicates were performed for this measurement. A t-test for independent samples was performed to compare the values of the two groups after four replications.

### Consumption of non-preferred food by starved and fed bees: grouped bees in a dual-choice situation

Bees captured at the hive entrance were placed in small cages (8.5 cm × 5 cm × 4 cm). All cages used were identical and all caged bee groups were similar in size (between 15 and 20 bees in all cases). The bees within a given cage belonged to the same colony. As they were not distinguished by task, they were subject to a homogenization period of the nutritional state in which a mixture of honey, pollen, sucrose and water was delivered during 3 h before starting the experiments. Cages were kept in cardboard box in the dark and at room temperature of 23 °C. Afterwards, they were divided in two groups subjected to two different feeding treatments: 1) fed bees had access to 0.6 M sucrose solution while and 2) starved bees had only access to distilled water *ad libitum*. Bees remained in their respective cage for 21 h (see Supplementary Fig. 1a).

At the end of this period, both starved and fed bees were presented in their corresponding cages (approximately 20 bees per cage) with one of three possible dual choice-situations (two different 1 mL Eppendorf tips pierced at their base, each with a different solution): 0.6 M sucrose solution vs. a mixture of 0.6 M sucrose solution and 10 mM quinine, 0.6 M sucrose solution vs. a mixture of 0.6 M sucrose solution and 100 mM salicin or 0.6 M sucrose solution vs. a mixture of 0.6 M sucrose solution and 3 M NaCl (Supplementary Fig. 1a). The amount of solution consumed was measured 3 h after offering this feeding choice by means of a 1 mL syringe; the amount consumed was the difference between the original volume of 1 mL and the remaining volume measured by the syringe. Consumption values were corrected for evaporation, which was measured in control cages^29^ which contained no bee, and expressed in terms of μl consumed per min per bee to facilitate comparisons between experiments.

### Consumption of non-preferred food by starved and fed bees: grouped bees in a single-choice situation

Bees captured at the hive entrance were placed in small cages (8.5 cm × 5 cm × 4 cm) and subject to the same treatment described for the previous experiment (see Supplementary Fig. 1b). At the end of the 24 h period, both starved and fed bees were presented in their corresponding cages (approximately 20 bees per cage) with one of four possible single feeding choices (two identical 1 mL Eppendorf tips pierced at their bases, both containing the same solution): either 0.6 M sucrose solution, or a mixture of 0.6 M sucrose solution and 10 mM quinine, or a mixture of 0.6 M sucrose solution and 100 mM salicin, or a mixture of 0.6 M sucrose solution and 3 M NaCl (Supplementary Fig. 1b). The amount of solution consumed was measured 3 h after offering the single feeding option as explained above. Consumption values were corrected for evaporation, which was measured in control cages^29^, and expressed in terms of μl consumed per min per bee to facilitate comparisons between experiments.

### Consumption of non-preferred food by starved and fed bees: individually-isolated bees in a single-choice situation

Honey bees captured at the hive entrance were enclosed individually in 5 mL syringes. The use of syringes imposed social isolation but restricted considerably the bees’ movements, thus preventing exhaustion. All syringes presented identical holes to allow for ventilation and respiration.

A 1 mL Eppendorf tip containing honey was inserted in the syringe tip so that the bee could access the food, which was generally consumed after 5 min. Bees were then divided according to two feeding treatments: fed bees received 0.6 M sucrose solution delivered by a 1 mL Eppendorf tip inserted in the syringe during a 4-h period. Starved bees received distilled water during the same period using the same method (see Supplementary Fig. 1c). After the end of this period, bees of both categories were divided in three subgroups (six in total) and presented during 2 h with one of three possible feeding options within their syringe via a new 1 mL Eppendorf tip: either a pure 0.6 M sucrose solution, or a mixture of 0.6 M sucrose solution and 10 mM quinine, or a mixture of 0.6 M sucrose solution and 100 mM salicin. The timing of this experiment was different from the previous ones as it was determined by the necessity of ensuring better survival in conditions (isolation within a small syringe) that were particularly stressful irrespectively of the food treatment delivered. The mixture of sucrose solution and NaCl was not assayed as no variations in its consumption were found between the dual-choice and the single choice experiments with caged bees (see above). The amount of solution consumed was measured and values were corrected for evaporation, as explained above, and expressed in terms of μl consumed per min per bee to facilitate comparisons between experiments. This form of expression was important as this experiment was shorter than the previous ones to ensure survival of the bees in particularly stressful conditions.

### Statistics

Analysis of variance (ANOVAs) was used in the food-choice experiments with individual rate of food consumption (μl/min/bee) as variable. In the dual-choice experiment, a repeated measurement ANOVA was used to determine whether the rate of consumption differed between the two feeding options delivered to each group of bees within each cage and between (across cages) feeding treatments. In the single-choice experiment, an ANOVA for independent groups was performed with feeding state (fed, starved) and feeding treatment (solution delivered) as factors. In the syringe-isolation experiment, a similar analysis was performed. In all cases, post hoc analyses were performed by means of Tukey tests and the alpha level was set to 0.05.

## Additional Information

**How to cite this article**: Desmedt, L. *et al.* Absence of food alternatives promotes risk-prone feeding of unpalatable substances in honey bees. *Sci. Rep.* **6**, 31809; doi: 10.1038/srep31809 (2016).

## Supplementary Material

Supplementary Information
