# Supplementary material for: Absence of food alternatives promotes risk-prone feeding of unpalatable substances in honey bees
Source: Sci Rep. 2016 Aug 18;6:31809. doi: 10.1038/srep31809 (PMC4989156; doi:10.1038/srep31809)

Absence of food alternatives promotes risk-prone feeding of unpalatable substances in honey bees

Lucie Desmedt[1,2], Lucie Hotier[1,2], Martin Giurfa[1,2], Rodrigo Velarde[3] and Maria Gabriela de Brito Sanchez[1,2] *

1: Centre National de la Recherche Scientifique (CNRS), Research Center on Animal Cognition (UMR5169), 118 route de Narbonne, 31062 Toulouse cedex 09, France

2: University of Toulouse (UPS), Research Center on Animal Cognition (UMR5169), 118 route de Narbonne, 31062 Toulouse cedex 09, France

3: Departamento de Biodiversidad y Biología Experimental, Grupo de Estudio de Insectos Sociales, Facultad de Ciencias Exactas y Naturales, Universidad de Buenos Aires, Pabellón II, Ciudad Universitaria (C1428EHA), Buenos Aires, Argentina

Supplementary Figure 1.

Experimental schedules of the three conditions in which consumption of non-preferred food was evaluated. **a)** Caged bees, starved and fed, subject to a dual choice situation. Bees had to choose between sucrose and a mixture of sucrose and an alternative non-preferred food such as quinine, salicin or NaCl. **b)** Caged bees, starved and fed, subject to a single-choice feeding situation. Bees had to feed on a single food type, either sucrose, a mixture of sucrose and quinine, a mixture of sucrose and salicin or a mixture of sucrose and NaCl. **c)** Bees, starved and fed, individually enclosed within a small syringe and subject to a single-choice feeding situation. Bees had to feed on a single food type, either sucrose, a mixture of sucrose and quinine or a mixture of sucrose and salicin.

Supplementary Figure 1

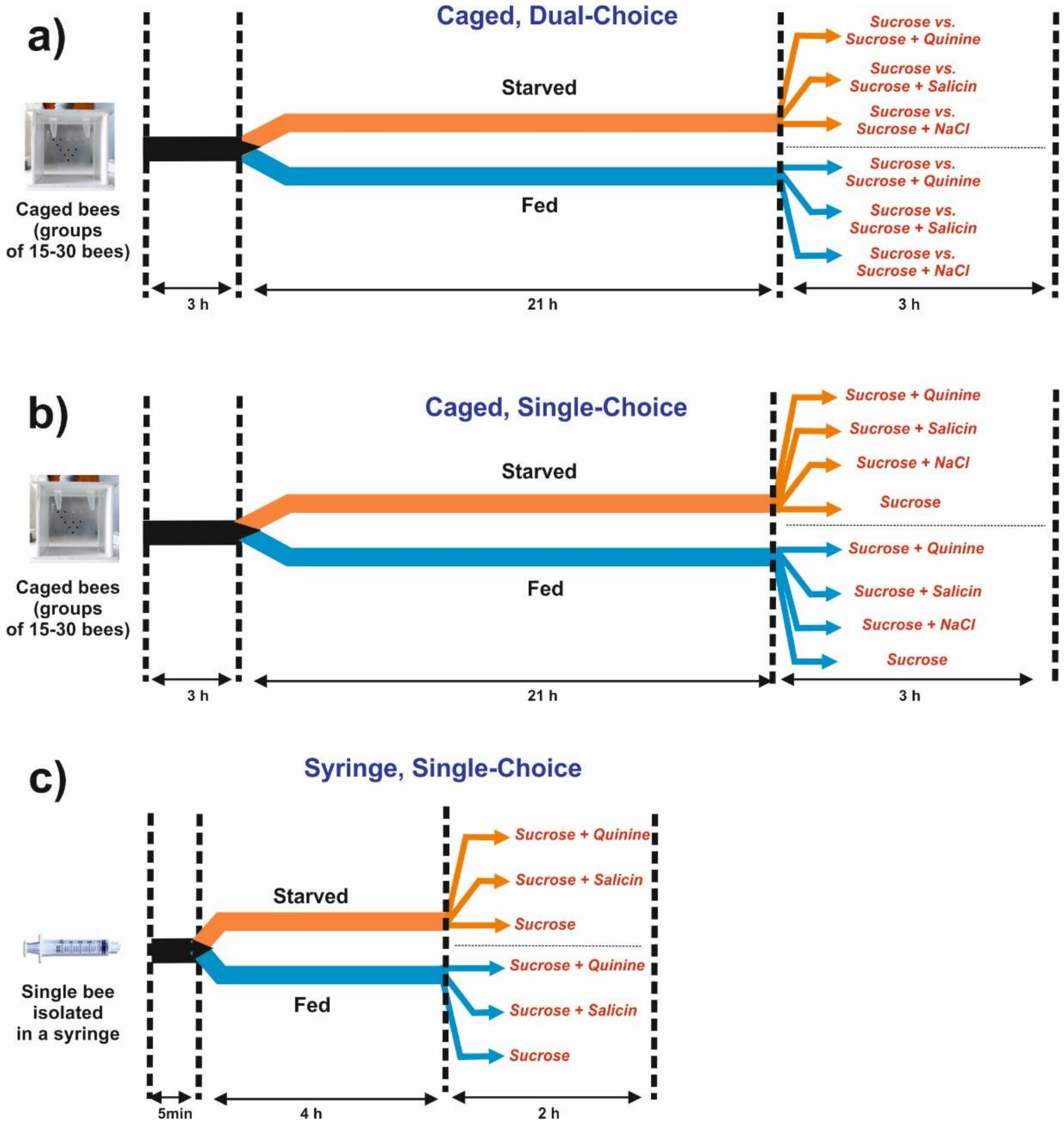

Supplement: Supplementary Information [file srep31809-s1.pdf]
